# Medical Society of the County of Erie

**Published:** 1911-08

**Authors:** Franklin C. Gram

**Affiliations:** Secretary


(Continued.)

### SOCIETY PROCEEDINGS

### Medical Society of the County of Erie

REPORTED BY FRANKLIN C. GRAM, M.D., Secretary

THE regular meeting of the Medical Society of the County of Erie was held in the rooms of the Buffalo Society of Natural Sciences on Monday evening, June 19, 1911, at 8.30 o'clock.

President McClure called the meeting to order and stated that, inasmuch as the minutes of the last meeting had been published in the Buffalo Medical Journal, their reading would be dispensed with unless otherwise desired. Hearing no objection, he so ordered.

The minutes of the Council meetings of June 5 and June 19 were then read and adopted.

Dr. Wall, chairman of the Committee on Membership, submitted names of applicants for membership, each of whom was separately ballotted for and elected, as follows:

Edward J. Ballou, Gardenville, N. Y.; George D. Stilson, 438 Potomac Avenue, Buffalo, N. Y.; Frank E. Lock, 226 Summit Avenue, Buffalo, N. Y.; Paul C. Campbell, 257 E. Delavan Avenue, Buffalo, N. Y.; Frank G. Walz, 905 Clinton Street, Buffalo, N. Y.; George A. Retel, 288 Broadway, Buffalo, N. Y.; E. G. Bodenbender, 660 Walden Avenue, Buffalo, N. Y.; Dr. Edward A. Sharpe, 162 Allen Street, Buffalo, N. Y., received by transfer from Medical Society of the County of Westchester.

Dr. Edward A. Sharpe, of Buffalo, presented a transfer from the Medical Society of the County of Westchester, stating that he was a member, in good standing, of said society. On motion, the transfer was accepted and Dr. Sharpe duly elected to membership in the Medical Society of the County of Erie.

Dr. George L. Brown, chairman of the Board of Censors, submitted report as follows:

"Mr. President and Members of the Erie County Medical Society: Your board of censors beg leave to submit the following report. At a meeting of the board of censors held May 6, Doctor Arthur G. Bennett, having resigned as chairman of the board, Dr. George L. Brown was elected to fill the vacancy. He received from the retiring chairman a bank check, amount $46.22 which, on the following day, he deposited in the Marine National Bank in the name of George L. Brown, chairman of the Board of Censors.

May 16, received the following letter, forwarded to me by the attorney of this Society:—

Chas. E. Doane, Esq.,

Dear Sir:—

Your letter of the 12th inst., relative to the appeal of John Treskow, charged with practicing medicine without a license, has been received. In reply, I beg to inform you that nothing has been done in this case, except a notice of appeal has been served by Cuddaback, Killeen and Karl, attorneys for the defendant. I am, therefore, unable at this time to inform you when this appeal will be argued.

Respectfully yours,
Clifford M. Laughlin,
Ass't District Attorney."

Receiving the following letter dated May 20—saying—"I beg to advise you, that I, as attorney for the Medical Society, obtained a warrant for Solomon Mesiroff, Proprietor of the Porter Medical Co., 333 Main Street. The warrant will be served as soon as Mesiroff returns to Buffalo."

That you may make a report of what has been done during the present year, I wish to advise you that one Consatino was convicted, on the 20th of January, 1911, of illegally practicing medicine. He was fined $25.00, and placed on probation for six months. I shall prepare a request for the payment of this fine to this Society.

Respectfully yours,
C. E. Doane.

A request has been prepared and presented."

We find the following names of physicians, who have registred at the County Clerk's Office since January 1, 1911.

We do not know to what date our predecessor made his last report.

Salvatore Bronchato, registered January 16. Born December 20, 1869, at Ventimiglin, Italy, located at 129 Court Street; A. S. Culkowski, registered March 11, born December 13, 1887, at Buffalo, New York, located at 94 Swinburne Street; John David Davis, registered January 26, born June 4, 1861, at Cassadaga, New York, located at Springville, N. Y.; Francis Hoehn, registered March 16, born at Niagara Falls, New York, January 13, 1888, located at 211 Amherst Street; John B. Hogan, registered March 17, born at Niagara Falls, New York, January 13, 1888, location not stated; George F. Harris, registered April 19, born February 6, 1874, at Binghamton, New York, located at State Hospital; Ralph R. Hughes, registered April 24, born at Amoki, Minnesota, April 12, 1886, located at 707 Crescent Avenue; Stephen P. Jewett, registered March 24, born September 1, 1883, at Waterford, Maine, located at 130 North Ashland Avenue; Julius J. Klein, registered April 18, born December 3, 1870, at Sandusky, Ohio, located at 343 Normal Avenue ;Alden D. Loomis, registered May 20, birth date and place not given, located at 6 East Seneca Street; Frank G. Lemon, registered May 22, born July 11, 1887, at New York City, N. Y., located at 400 Forest Avenue; Erwin H. Mudge, registered March 10, born December 14, 1886, at Olean, New York, located at 822 Elmwood Avenue; Isaac D. F. Nichols, registered December 27, 1910, born April 14, 1845, at New York City, N. Y., located at 6 East Seneca Street; Oreon H. Rhodes, registered March 15, born January 31, 1865, located at 3 Brisbane Building. He is advertising in the Press of this City, and New York City and Pennsylvania exclusively, to cure rupture and hemorrhoids; Frederick P.

Schenkelberger, registered April 24, born February 3, 1887, at Hiram, Ohio, located at Gowanda, N. Y.; Harry Sickerman, register May 16, born October 30, 1885, at Austria, located at 316 North Division Street; Isaac Weinstein, registered March 13, born December 25, 1867, at Koono, Russia, located in Buffalo, N. Y.

May 7th swore out a warrant for the arrest of Fannie Ciresa for practicing medicine illegally. She was located on the Terrace. She was advertising in the Italian papers as a Mechano-Terapist. The warrant has not been served as Miss Ciresa evidently became acquainted with the fact and has left the city.

June 8, 1911, swore out a warrant for the arrest of Lucius Auld, located at 79 Niagara Street, for practicing medicine without being registered in the Board of Health office. He is a successor to Dr. Walker, the advertiser. June 9, Doctor Lucius Auld plead guilty and paid the court $10.00.

June 9, swore out a warrant for the arrest of Abraham Simon residing at 232 Mortimer Street, for vaccinating children without having a license. June 16, he plead guilty and paid the court a fine of $50.00.

June 12, 1911, Solomon Meseroff, residence Chicago, Ill., was arrested for practicing medicine illegally, being the owner of the Porter Medical Co., removed recently from the corner of Main and North Division Streets to the corner of Main and South Division Streets. This case was called in Judge Hartzell's Court and adjourned to June 14, 2.30 p. m. On that date the case was partly tried and then adjourned to June 29. Mr. Meseroff is out under bail of $500.00.

Signed by
GEORGE L. BROWN, Chairman
EDWARD CLARK,
A. G. BENNETT,
FRANCIS C. FRONCZAK,
LAWRENCE HENDEE, Secretary.
Board of Censors.

The report was accepted and approved and the thanks of the Society offered to the Board of Censors, especially its chairman.

Dr. Mulford, chairman of the Committee on Necrology, read memorials on the deaths of Drs. Frank B. Seitz, William Warren Potter, George Abbott and Carlton C. Frederick.

The memorials were adopted and ordered filed.

Dr. Edward E. Haley, chairman of the Committee on Contract Work, presented a very exhaustive report of his committee.

Dr. Wall moved that the report be approved and the resolutions contained therein adopted.

Dr. Crego moved, as an amendment, that contracts be not renewed after January 1, 1912.

Dr. Haley explained that contracts expire at various seasons and not all at the beginning of the year.

Dr. Crego withdrew his amendment with the understanding that this matter would be further considered, the amendment, proposed by Dr. Haley in his report, to lie on the table, under the rules.

Dr. Brown moved that Dr. Haley's report be published in the Buffalo Medical Journal and the New York State Journal. Motion was carried.

On motion of Dr. Wall, the committee will be continued, and it was requested that further report be made at the October meeting.

Dr. Wall then moved to take from the table his amendments to the by-laws, which had been tabled at the December meeting.

On motion of Dr. Wall, amendments were adopted as follows:

Amend Chapter 11, Sec. 1—The Medical Society of the County of Erie, being an integral part of the Medical Society of the State of New York, and the District Branch, membership may be obtained, etc.

Amend Chapter 11, Sec. 5—Candidates duly elected shall qualify as members by signing a statement that they will be governed by the by-laws of this Society.

Amend Chapter 5, Sec. 2—Insert between the word Society and appropriated, the words, "as authorized by these by-laws and as" and substitute for words "Estimates of the amounts needed," —the words "a report and recommendation of action necessary."

On motion of Dr. Grosvenor, who opposed it, the following was referred to Council for reconsideration:

Amend Chapter 8, Sec. 4—Add after co-operate with, the words, and take no action on State Legislation or appointments until approved by."

Dr. Wall moved to substitute "President" for "Council" in Sec. 2 of Chap. VIII.

Dr. Grosvenor objected to the entire section and asked whether it was intended to eliminate the present section. He then moved to insert, after the word "delegate," the words "to the house of delegates," and the section was adopted as follows:

Chapter 8, Sec. 2—In case a delegate to the house of delegates cannot attend any meeting, the president shall appoint an alternate, and if any representative fails to appear, the other delegates present shall choose a member to act for him."

The following were then adopted:

Amend Chap. XI, Sec. 1—Insert "or" before "as arranged."

Amend Chap. XI. Sec. 5—to read, The order of business when not arranged by the council and printed in the notice of the meeting, shall be, and the like.

Amend Chap. XV. Sec. 1—Insert between words "been" and "sent" "approved by the council of the State Society and advice of the proposed change."

Dr. Grosvenor moved to substitute the word "at" for the word "for" in Sec. 2 of Chap. XV, making the section read as follows:

Chap. XV. Sec. 2—Any by-law except Chapter XV, Sects. 1 and 2, may be suspended at any meeting, and the like.

Section was then adopted.

Dr. Lytle stated that at the annual meeting he had been directed to prepare an amendment to the by-laws, governing the sinking fund, and he offered the following:

Amendments to by-laws, creating and defining a Sinking Fund, which were ordered prepared at the annual meeting December 19, 1910.

Amend Chapter XII so that caption shall read "Dues and Finances" instead of "Dues."

Amend by adding to Chapter XII two sections to be known as sections 4 and 5 which shall read as follows:

Sec. 4—After paying all outstanding floating indebtedness, all money derived from dues and in excess of one hundred dollars ($100.00), that remains in the treasury at the close of each year (December 31), as well as such other money as this society may from time to time direct, shall be deposited in savings institutions designated by the Council, to the credit of a fund to be known as the Permanent Fund of the Medical Society of the County of Erie.

Sec. 5—No money shall be withdrawn from the Permanent Fund of the Medical Society of the County of Erie except by a two-thirds vote of the members present at a regular meeting, and provided that notice of intent to move for such withdrawal shall have been approved by the Council and a copy thereof sent to each member with the notice for the meeting at which such withdrawal will be considered.

Under the rules, this amendment will lie on the table.

Dr. Lytle made a report of the expenditures for the McCormick meeting which amounted to $30.34 as the Society's share, but since that time, $5.00 have been returned to the Society. Report was approved.

The secretary read a communication from the Medical Society of the County of Dutchess, as follows:

Dr. Franklin C. Gram.
Secretary of the Medical Society of the County of Erie.

Dear Doctor:

At the regular meeting of the Medical Society of the County of Dutchess, held April 12, 1911, the following resolutions were adopted:

WHEREAS, We consider it impracticable and impossible for the average sized County Medical Society to secure the enforcement of the laws regulating the practice of medicine;

*Resolved,* That our delegates to the State Society be directed to bring this matter to the attention of the House of Delegates, with the intent that the enforcement of such laws be assumed by the State Society;

*Resolved,* That the Secretary send copies of these resolutions to each of the County Medical Societies in this State.

Trusting that you will bring this matter before your Society, I am

Very truly,

FREDERICK J. MANN,

Secretary.

June 5, 1911.

These resolutions were opposed by everyone present, for the reason that the Board of Censors is better able to look after such work than the State Society; Violators of the law being known in the County where the violations occur, the work can be done cheaper and more thoroughly than if entrusted to the State Society which would necessarily be under a great expense in trying to enforce the law. On motion our Delegates to the State Society were directed to oppose this proposition.

A letter was received from the Health Commissioner of Buffalo and read as follows:

June 19, 1911.

Dr. Daniel V. McClure,
President of the Medical Society of the County of Erie,
Buffalo, N. Y.

Dear Dr. McClure:

At the meeting of the Board of Aldermen held today, a resolution was introduced looking to the rescinding of the ordinances providing for vaccination of teachers and pupils in the public schools of our city, and the like, the apparent reason for this action being the death, from general septicemia following vaccination, of a boy who had been vaccinated. The department tried to offset this matter, and I personally appealed to the editors of our daily papers to not publish anything about it, so as not to unnecessarily alarm the public.

They insisted upon the publication, however, and I, therefore, explained regarding the infection in order, if possible, to belittle the rumors and statements that vaccination was the cause of death.

A public hearing on the proposed rescinding of the ordinances has been set for Thursday night of this week, and I feel it quite necessary that a large committee be appointed to represent the Society in this matter and take such part in the hearing as may be deemed expedient and proper.

Respectfully yours,
FRANCIS E. FRONCZAK,
Health Commissioner.

Dr. McKee moved that the President appoint a strong committee in compliance with Health Commissioner Fronczak's request. Motion was adopted.

Dr. McKee moved that a committee be appointed to confer with the department of health, with a view of selecting a more appropriate time, for the vaccination of school children, than the end of the school year. Motion was adopted, and the President appointed Dr. McKee as chairman of such committee.

Assistant Health Commissioner Schaefer explained that the time for vaccination had been set at that period at the request of the School Principals of Buffalo.

Treasurer Lytle stated that the by-laws require payment of dues before April 1st of each year, and that the treasurer, under the by-laws, was directed to present the names of all those who are in arrears, at the next meeting of the Society.

This being the next meeting after that date, he felt compelled to make a report, which he did.

Dr. Joseph C. O'Gorman, a member of the Entertainment Committee, announced that this year's outing would be held at Island Park, Grand Island, July 25, boats to leave the foot of Ferry Street every hour; dinner from 5 to 6 o'clock; tickets, including boat ride, dinner and cigars, $1.50. He requested everyone to supply himself with tickets and to support the outing with his presence.

This ended the business session and Dr. W. W. Quinton read a brief but interesting paper on the Treatment of Syphilis.

Dr. Roland O. Meisenbach then presented "An apparatus for the measurement of spinal curvature with method of recording cases."

After a brief discussion of the subjects presented, the meeting adjourned and a collation followed.

Franklin C. Gram,
Secretary.

At the regular meeting of the Medical Society of the County of Erie, memorials of Drs. Frank B. Seitz, William Warren Potter, George Abbott and Carlton C. Frederick were presented by the Committee on Necrology, through its chairman, Dr. Henry J. Mulford. These are given in brief, as follows:

Frank Burghardt Seitz, born at Rochester, N. Y., June 2, 1862. His preliminary education was obtained in the public schools of Rochester; his medical education at the Hahnemann Hospital at St. Louis, from which he graduated in 1891. For five years he was engaged in general practice at Rochester, during which period he was connected with the Rochester Homeopathic Hospital. In 1897 he went abroad for special study and on his return to America he opened an office at 33 North Pearl Street, Buffalo, for special practice in diseases of the eye, ear, nose and throat. Later he removed to 21 North Street, where he lived up to the time of his death.

Dr. Seitz was a member and a vice president of the Western New York and State Homeopathic Societies; a member of the Clinical Club of Buffalo; of the Buffalo Academy of Medicine, and Medical Society of the County of Erie. He also was a member of Holy Trinity Church, and of Washington Lodge F. and A. M.

Dr. Seitz was the author of various professional papers, which he had read before the medical societies to which he belonged; and of a book, "Through Sixteen European Cities," which he wrote after his trip abroad.

In January, 1910, Dr. Seitz contracted pneumonia, which left him in poor general condition and he never fully regained his health. He died June 14, 1910, at the age of 48, leaving a wife and five children.

—

Any man who has lived fifty-two years at the service of his profession deserves the kindest thought of that profession, the highest honors in its gift. Such a man was William Warren Potter. His medical life covered a period longer than the entire life of the average man; and, during that period, his labor was for his profession. His aim was its advancement, not the advancement of himself through it. His life illustrates this. He was ever ready to lend a helping hand to the younger members of his profession (there are many in Buffalo who can testify to this); and he died without riches. Every man has his faults, but in William Warren Potter there is no fault broad enough to blot the record. Truly, we may say that he was a gentleman, a gentleman at the service of his profession.

William Warren Potter was born at Strykersville, N. Y., December 31, 1838, the son of Dr. Lindorf and Mary Green

(Blanchard) Potter. He received his academic education at Arcade Seminary, N. Y., and at Genesee Seminary and College, Lima, N. Y. His medical education was obtained at Buffalo University Medical College, from which he graduated in 1859.

At the beginning of the civil war, two years later, Dr. Potter offered his services to the government and served three years with the Army of the Potomac. For "faithful and meritorious service" he was brevetted Lieutenant Colonel of United States Volunteers by the President of the United States, and Lieutenant Colonel of New York Volunteers by the Governor of the state of New York.

Returning to civil life Dr. Potter soon located at Buffalo, limiting his practice to diseases of women. In July, 1888, he became editor of the BUFFALO MEDICAL JOURNAL and shortly thereafter owner as well. He was a member of the several local, state and national societies, examiner in obstetrics and gynecology of the New York state medical examining board and president since 1897. He was a companion of the Military Order of the Loyal Legion of the U. S.; and a member of Bidwell-Wilkinson Post, G. A. R.

Dr. Potter died at Buffalo on March 14, 1911, after a month's severe illness.

The funeral services were held at 238 Delaware Avenue on March 16, the Reverend Cameron J. Davis, rector of Trinity Church officiating.

Dr. Potter's only son, Dr. Frank Hamilton Potter, died in 1891.

—

GEORGE ABBOTT was born at Palmyra, Wayne County, N. Y., November 2, 1826, was a descendant of George Abbott of Rowley, Mass., who came there from England in 1642. His father, the Reverend Orrin Abbott, was a minister, a soldier in the war of 1812, and a Chaplain of the 98th regiment N. G. S. N. Y. 1864.

Dr. Abbott attended the Akron High School and the Geneva Medical College, completing his medical course at the Buffalo Medical School, in February 1852. During his resident service at the Buffalo Hospital of the Sisters of Charity, he contracted Asiatic cholera near the end of the epidemic.

April 1, 1853, Dr. Abbott opened an office at Hamburg, N. Y. In 1854 he was appointed surgeon of the 67th Regiment N. G. S. N. Y. In 1856 he was elected Town Superintendent of schools, and helped to organise the Hamburg Union School and Academy. In 1872 he was elected School Commissioner, serving two terms. From 1857 until 1860 diphtheria was epidemic in Western New York, and he took a leading position in treating the disease, and with marked success. A paper which he afterward wrote on this subject was copied by the London Medical Journals.

In 1863 Dr. Abbott was mustered into the service of the United States in response to a call for militia to assist in repelling General Lee's attempted Northern raid, and served forty-three days. He was then appointed by the Governor of New York State to raise a regiment of militia for the Third assembly District of Erie County. Within sixty days he had raised seven companies of thirty-two men each, which were organised as the 98th Regiment N. G. S. N. Y., and he was made its Colonel. On August 10, 1864, the regiment was mustered into the service of the United States, doing guard duty at Elmira, over 15,000 prisoners of war during more than four months.

Dr. Abbott was a member of the old Erie County Medical Society, having acted on its board of censors, as its vice-president in 1864-'65, and its president in 1866. He was a member of the G. A. R.; past member of Zion Lodge, F. and A. M. of Orchard Park, N. Y., and, for many years, of Fraternal Lodge No. 625, F. and A. M. of Hamburg. At one time he was treasurer of the New York State Grange. In politics he had been an Anti-Slavery Whig, later becoming a Republican.

Dr. Abbott was married to Julia Clark Church, of Fort Ann, Washington County, at Hamburg, April 19, 1857. Mrs. Abbott, and two of their five children, George Burwell Abbott of Hamburg, and Mrs. Peter Thompson of Spencerport, N. Y., survive him.

Dr. Abbott had been in failing health for some years previous to his death, which occurred on March 26, 1911. The funeral was on March 30th, from the Methodist Church, and burial was in the Hamburg Cemetery.

---

Carlton Cassius Frederick was born at Hamburg, N. Y., May 1, 1855. His parents were Peter C. Frederick and Mary Jane Clough, his wife. When he was fourteen the family removed to Buffalo where he attended Grammar School No. 14, and the Central High School. He received the degree B. S. from the University of Michigan in 1877. During 1877-'78 he taught school at North Evans, N. Y., and during 1878-'79 he taught in Professor Briggs' Classical School. In 1879 he entered the Buffalo University Medical School, graduating from there in 1881.

Dr. Frederick was interne at the Buffalo General Hospital for the year following his graduation. Then opening an office at 64 Richmond Avenue, he devoted himself to general practice. From the first he was successful, developing one of the largest general and obstetrical practices in Buffalo. As his experience grew he gave his attention more and more to abdominal and pelvic surgery, until, at the time of his death, he had become an authority in that branch of his profession. Both his consultation and his operative work were very extensive, not only in Buffalo itself, but also throughout the surrounding territory.

Dr. Frederick was associated with Dr. Thomas Lothrop when the latter started a Maternity Hospital at Seventh and Maryland Streets, and, later, in the founding of the Women's Hospital. The first abdominal operation performed in the hospital was done by Dr. Frederick on November 19, 1891, assisted by Dr. W. S. Tremaine. The case was one of double pyosalpinx.

In 1891 Dr. Frederick went to Europe and studied under Martin and Olshausen at Berlin; Sanger at Leipsic; Leopold at Dresden; and Lawson Tait in England.

In 1885 Dr. Frederick was obstetrician to St. Mary's Asylum in Edward Street; in 1908 President of the Medical Society of Central New York. He was a Member of the Alumni Association of the University of Michigan; of the Medical Society of the County of Erie; the Medical Society of the State of New York; the American Medical Association; the Buffalo Medical Club; the Buffalo Academy of Medicine, and the Alumni Association of the Buffalo General Hospital. He also was a Member of the American Gynecological Society and the American Association of Obstetricians and Gynecologists. He was Adjunct Professor of Obstetrics in the Medical Department of Niagara University, and, later, Clinical Professor of Gynecology in the University of Buffalo.

Dr. Frederick was President of the Buffalo Academy of Medicine at the time of his death.

In 1885 Dr. Frederick married Elizabeth B. Smith of Syracuse, N. Y., who survives him, together with one son, Thomas Lothrop Frederick, and one daughter, Ruth.

Dr. Frederick died on April 30, 1911, aged 56 years, after a brief illness, death being due to endocarditis.

---

Dr. John Sanford Halbert, of 2485 Main St., died at Rochester, Minn., July 18, 1911. Dr. Halbert was a graduate of the University of Buffalo, 1872. He held the position of Obstetrician to the Homoeopathic Hospital.

